# “I Think I Am Getting There” Understanding the Computational Identity of Engineering Students Participating in a Computationally Intensive Thermodynamics Course

**DOI:** 10.1007/s43683-022-00084-1

**Published:** 2022-09-07

**Authors:** Huma Shoaib, Aasakiran Madamanchi, Elsje Pienaar, David M. Umulis, Monica E. Cardella

**Affiliations:** 1 School of Engineering Education, Purdue University, West Lafayette, IN USA; 2 School of Information, University of Michigan, Ann Arbor, MI USA; 3 Weldon School of Biomedical Engineering, Purdue University, West Lafayette, IN USA; 4 School of Universal Computing, Construction, and Engineering Education (SUCCEED), Florida International University, Miami, FL USA

**Keywords:** Computational identity, Engineering identity, Gender

## Abstract

In response to the growing computational intensity of the healthcare industry, biomedical engineering (BME) undergraduate education is placing increased emphasis on computation. The presence of substantial gender disparities in many computationally intensive disciplines suggests that the adoption of computational instruction approaches that lack intentionality may exacerbate gender disparities. Educational research suggests that the development of an engineering and computational identity is one factor that can support students’ decisions to enter and persist in an engineering major. Discipline-based identity research is used as a lens to understand retention and persistence of students in engineering. Our specific purpose is to apply discipline-based identity research to define and explore the computational identities of undergraduate engineering students who engage in computational environments. This work will inform future studies regarding retention and persistence of students who engage in computational courses. Twenty-eight undergraduate engineering students (20 women, 8 men) from three engineering majors (biomedical engineering, agricultural engineering, and biological engineering) participated in semi-structured interviews. The students discussed their experiences in a computationally-intensive thermodynamics course offered jointly by the Biomedical Engineering and Agricultural & Biological Engineering departments. The transcribed interviews were analyzed through thematic coding. The gender stereotypes associated with computer programming also come part and parcel with computer programming, possibly threatening a student's sense of belonging in engineering. The majority of the participants reported that their computational identity was “in the making.” Students’ responses also suggested that their engineering identity and their computational identity were in congruence, while some incongruence is found between their engineering identity and a creative identity as well as between computational identity and perceived feminine norms. Responses also indicate that students associate specific skills with having a computational identity. This study's findings present an emergent thematic definition of a computational person constructed from student perceptions and experiences. Instructors can support students’ nascent computational identities through intentional mitigation of the gender stereotypes and biases, and by framing assignments to focus on developing specific skills associated with the computational modeling processes.

## Introduction

Most engineering fields are becoming more computationally intensive because of the growing demand from industry for students to be able to use computers and programming skills to develop computational models. One of the fields rapidly adapting to this increasing computational intensity is biological engineering.^42^ Within biological engineering, and biomedical engineering, it is becoming increasingly common for engineers to use computers to create code to construct and analyze a model of an organ, a cell or a bioreactor; and use the model to make engineering decisions. As students are finishing their degrees and applying for jobs, they might see positions advertised as “computational biologist” or “computational analyst”. At the same time, we are seeing a growing number of graduate programs with a “Computational Biological Engineering” focus (e.g. Carnegie Mellon University).

However, there is limited engineering education scholarship on how best to support students in learning computationally intensive approaches to understanding and solving biological and biomedical engineering problems. In our own prior work, we have found that learning how to translate biological phenomena into a computational model can be more challenging for learners than learning how to code. More than just being the application of mathematics or programming knowledge, computational modeling requires problem decomposition and abstraction, two key components of computational thinking.^28,50^ In the absence of research-based strategies for integrating topics focused on creating and using computational models into biological engineering and biomedical engineering curricula, the increased computational intensity in biomedical engineering curricula may be ineffective or even counterproductive.

One area of concern is gender parity. While biomedical engineering and biological engineering have higher participation rates of women than other engineering disciplines, there is still a precipitous drop in representation at the graduate and faculty levels and in specific areas like computational biology.^6^ Additionally, as biomedical engineering and biological engineering disciplines rely more heavily on computing for simulation and data analysis, students perceive that computational ability is an increasingly important skill needed in their discipline. It is essential to understand if the growing demand for computational skills impacts women's participation in engineering. Lichtenstein* et al*.^26^ argue that the problem of underrepresentation of women in engineering programs begins with socio-cultural factors such as gender stereotypes, subtle biases against girls in early education, lack of encouragement and exposure to mentors, and essentialist thinking where men are believed to be inherently good at computational skills. There is a long-enduring stereotype that women are inherently unsuited to be programmers, which can lead to women feeling that they do not belong, having less confidence, or having less motivation to engage with computational project work.^39,45^ Understanding the experiences of women in computationally-intensive biomedical engineering and biological engineering courses is critical for sustaining and increasing the participation of women in Biomedical engineering as the field continues to become more computationally intensive.

Crucially, in considering gendered experiences in engineering and computing, we recognize that gender is more than binary. However, in this paper we focus primarily on women. We make this choice because the population of students enrolled in our course identified as either men or women (and the students did not disclose whether they were cis or trans). Thus, we did not have the opportunity to attempt to interview students who identified as non-binary or another gender identity.

Gender is of importance in considering engineering and computational identity because of gender disparity issues in engineering and computational disciplines.^22^ While there has been some progress towards gender parity in sciences, with women earning almost 44% of undergraduate degrees in STEM in recent years,^35^ women still are underrepresented in specific fields, such as engineering and computer science.^36^ Computer science and most fields of engineering remain male-dominated fields.^11^ Women’s perceptions of STEM fields as masculine fields because of the gender imbalance have resulted in women being less likely to report long-term career plans in fields like engineering.^2^ To address and understand retention and persistence in engineering, many researchers have used engineering identity to investigate factors that contribute to students’ decisions to participate and persist in engineering.

### Computational Identity and Other STEM Identities

Identity is an essential construct in understanding student persistence, the process of learning, and a way to promote equity in STEM.^15^ As people have studied identity, researchers have taken many different approaches and different theoretical framings. We build on earlier work that recognizes that identity is not necessarily stable, but instead changes and evolves through a series of experiences and through interactions with others, and that individuals can hold multiple identities at once.^8,48^ Identity refers to the roles of a self, constructed by meanings that a person attaches to the many roles they play in their world.^46^

According to *social identity* theory, when an individual holds an identity, they will act based on that acquired identity and align their action(s) with the community/social group they are participating in to achieve their goals. This framing of identity helps us understand how identities are maintained and manifested in social interactions. This theoretical framing of identity has been adapted by researchers working in engineering identity, where Godwin* et al*.^17^ adapts social identity theory to describe group identities like gender, but role identity theory to describe taking on what it means to be an engineer.

**Figure 1:**
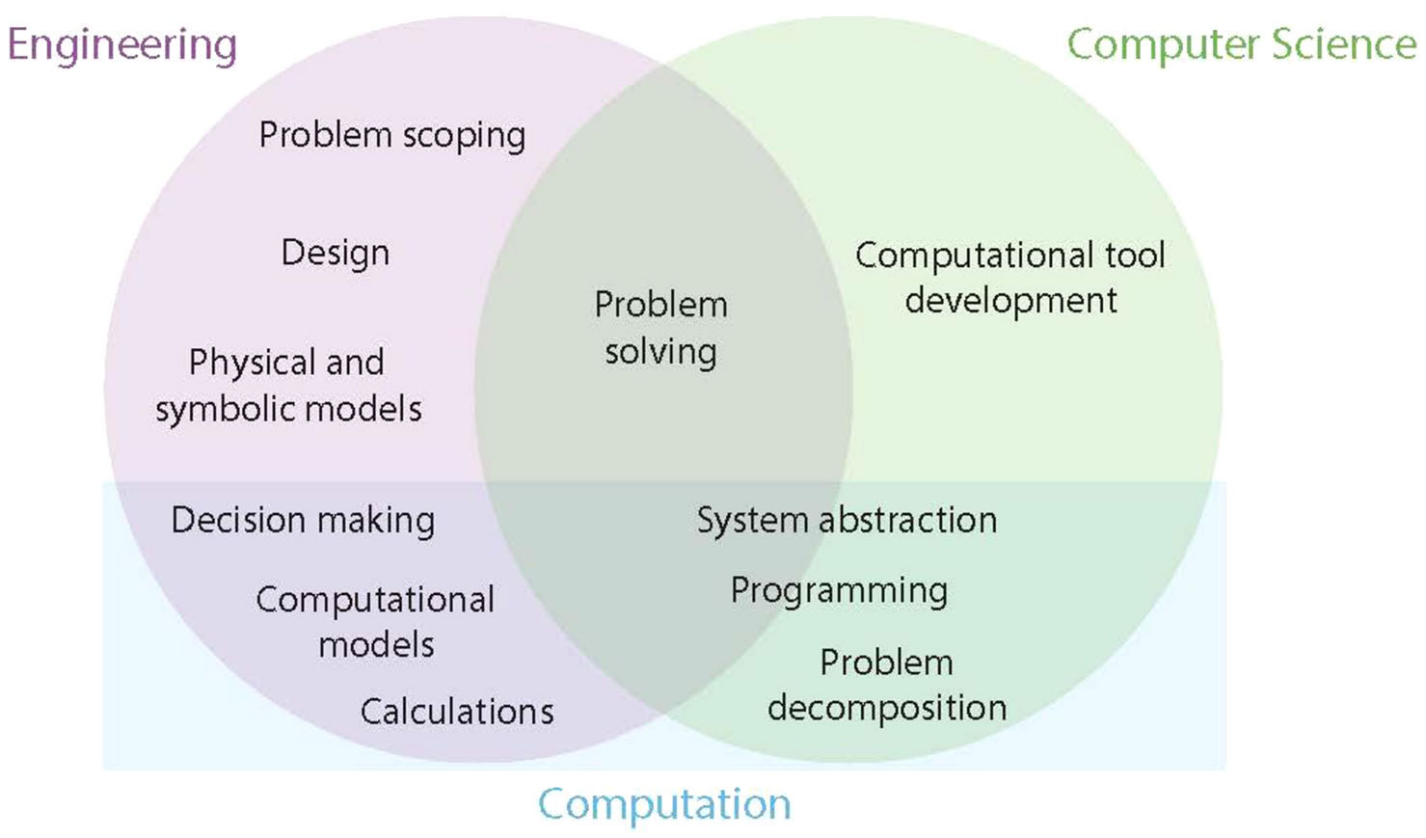
Relationship between engineering, computation, and computer science.

Recently, many STEM education researchers have examined identity development through a *disciplinary identity* framing. With this framing, researchers consider how people develop identity with respect to a specific discipline. Three dimensions of this framework contribute to the development of a disciplinary identity: students’ sense of whether they are capable of performing within the discipline, students’ interest in the discipline, and students’ feelings of recognition by others within the discipline.^20,30^ This framing has the potential to be used to understand disciplinary identity in many different disciplines, where the focus on performance, interest, and recognition remains consistent, but the context for the performance, interest, and recognition varies. For our consideration of computational identity, we draw on work on engineering identity and computer science identity, as a computational identity for an engineering student would likely have some overlap with these two areas (see Fig. 1 for our interpretation of the relationship between engineering, computation, and computer science).

One of the identities that we anticipate that undergraduate engineering students might develop is an engineering identity. Some studies that consider engineering identity as involving seeing oneself in the role of an engineer have explored this by conceptualizing engineering identity as the knowledge, emotions, abilities, and experiences surrounding one's roles as an engineer,^13^ and by examining the configuration of roles an engineer assumes in the workplace, which varies based on the environment.^19^ Mann* et al*.^31^ consider engineering identity as a combination of an individual having a self-belief about being an engineer and others recognizing the individual as an engineer at the same time. Tonso^47^ noted that when someone is referred to as an engineer in everyday settings, it signals to the individual being referred to as having an engineering identity because “the individual” belongs to a community of engineers. Similar to the framing for this study, Rodriquez* et al*.^40^ defined engineering identity as a measure of an individual’s perception of their recognition as an engineer by others, competence as an engineer, and interest in engineering. Across these studies, engineering identity involves seeing oneself in the role of being an engineer, being recognized as an engineer by others, participating in the community of engineers, and having the abilities and knowledge to perform engineering.

While there is a growing abundance of literature on engineering identity, the literature on computational identity in undergraduate engineering is nearly nonexistent. Because of the paucity of research on computational identity amongst undergraduates we look to the broader STEM education literature. In a recent study Kong and Wang^25^ conceptualized “Computational Identity as an ongoing mental construction process of self-identification resulting from total immersion in feelings and experiences of programming activities at school.” More specifically, the study conceptualized Computational Identity with the following four components: (1) programming engagement, (2) programming affiliation, (3) programming actualization, and (4) programming goal setting. They report that Computational Identity is not necessarily fostered by learning computing skills and requires explicit consideration in instructional design. Kong’s definition of computational identity is constructed through computational thinking^7^ and social identity theory^23^ frameworks for a project focused on children in the 4th–6th grade (approximately 9–11 years old). However, we note several limitations in how Kong and Wang’s conceptualization of Computational Identity. First, investigation is needed to better understand computational identity in higher education. Second, Kong and Wang’s decision to base computational identity on social identity was appropriate given their focus on children, where there is an overall focus on children developing knowledge, skills, and self-efficacy instead of choosing disciplines to study as an undergraduate major or participate in as a professional. However, to study how computational thinking might contribute to engineering persistence for undergraduates, we believe it is important for an identity framework to consider disciplinary identity in addition to social identity. Understanding the experiences of undergraduates in computational fields and how they associate their identity with these experiences is crucial for attracting and retaining students in engineering and computational programs. Finally, Kong and Wang’s conceptualization of Computational Identity is limited to a focus on programing and does not include other aspects such as the application of mathematics; development, interpretation and use of computational models; and aspects of computational thinking (e.g. problem decomposition and abstraction).

While computational identity is distinct from a computer science or computing identity, recent studies of the identity development of computer science students can inform how we think about a student’s computational identity. Some identity studies have looked at how computer science students perceive their engagement in computer science and information technology (IT) as meaningful. Identity plays a key role in the process of defining a career path as well as engagement in computing activities in university.^38^ Garcia * et al*.,^15^ report recent work on computing identity within the context of undergraduate computer science, computer engineering and information technology. Their findings shed light on students’ self-perceptions related to three dimensions of disciplinary identity: recognition, interest, and performance/competence. The findings from the Garcia* et al*. study suggest that even amongst high achieving students, women participants had less of a computing identity than men. The Garcia* et al*. study also advances a framework of computational identity based on disciplinary identity that is further described in a paper by Mahadeo* et al*.,^30^ we discuss this framework further in a later section on our Theoretical Framing for this study. In another recent study, Kapoor and Gardner-McCune^24^ conducted research on computer science undergraduate students and how these students identify themselves professionally. Kapoor and Gardner-McCune define computing professional identity as transformation of interest in computing to self-perception of engaging with computing as a career. Kapoor and Gardner-McCune’s study reports that these students explored computing professions through their involvement in professional development activities, informal activities, coursework and negotiations with people in the broader community. However, computer science identity or computing identity was not clearly defined. For undergraduates, the work on computing identity is limited to only computer science, software engineering, and information technology undergraduate students. Little work has been done to understand and investigate the experiences of undergraduate students who are participating in computational activities outside computer science and information technology in engineering.

### Gender and Computational Identity

Recent reports and papers establish the prevalence of gender stereotypes in computing disciplines. For example, stereotypes of ‘geeks’ and ‘anti-social nerds’ are prevalent stereotypes of computing students^33,51^ and are recognized in computing as possible causes of underrepresentation of women in computing disciplines. When students see “antisocial” as a defining characteristic of a computing person, they may see themselves as someone who is able to do computing, while at the same time not see themselves as a computing person,^51^ and then start to lose interest or feel like they do not belong. Computing has stereotypical characteristics (e.g. antisocial behavior and nerd stereotypes) and both men and women who want to depict acceptance in this masculine field adapt to these characteristics.^12^ If someone sees a domain or role as congruent with self, they can easily develop an identity. However, in their study, Peters and Pears^38^ found that many undergraduate students’ perceptions of the computing field were misaligned with their personal identities and values. When there is incongruence between a domain or role and a person’s sense of self, it causes identity conflict that can lead to coping strategies to minimize one’s identity in a particular context to create fit or to leave.^34^

### Theoretical Framing

This study adapts the framing of identity which comes from social identity theory^46^ and symbolic interactionism.^9^ As noted earlier in the paper, social identity theory helps us understand how identities are maintained and manifested in social interactions (e.g. in classroom environments, when students are working on projects in teams). Symbolic interactionism is the meanings that students develop as a part of social interaction—in this case, in the computationally intensive engineering course. This study also builds on the disciplinary identity framework that has been used in computer science education research for understanding computing identity,^30^ where three primary sub-constructs that contribute to the development of a disciplinary identity: belief in one's performance/competence, interest in the discipline, and recognition by others that one has expertise relevant to the discipline.

**Figure 2:**
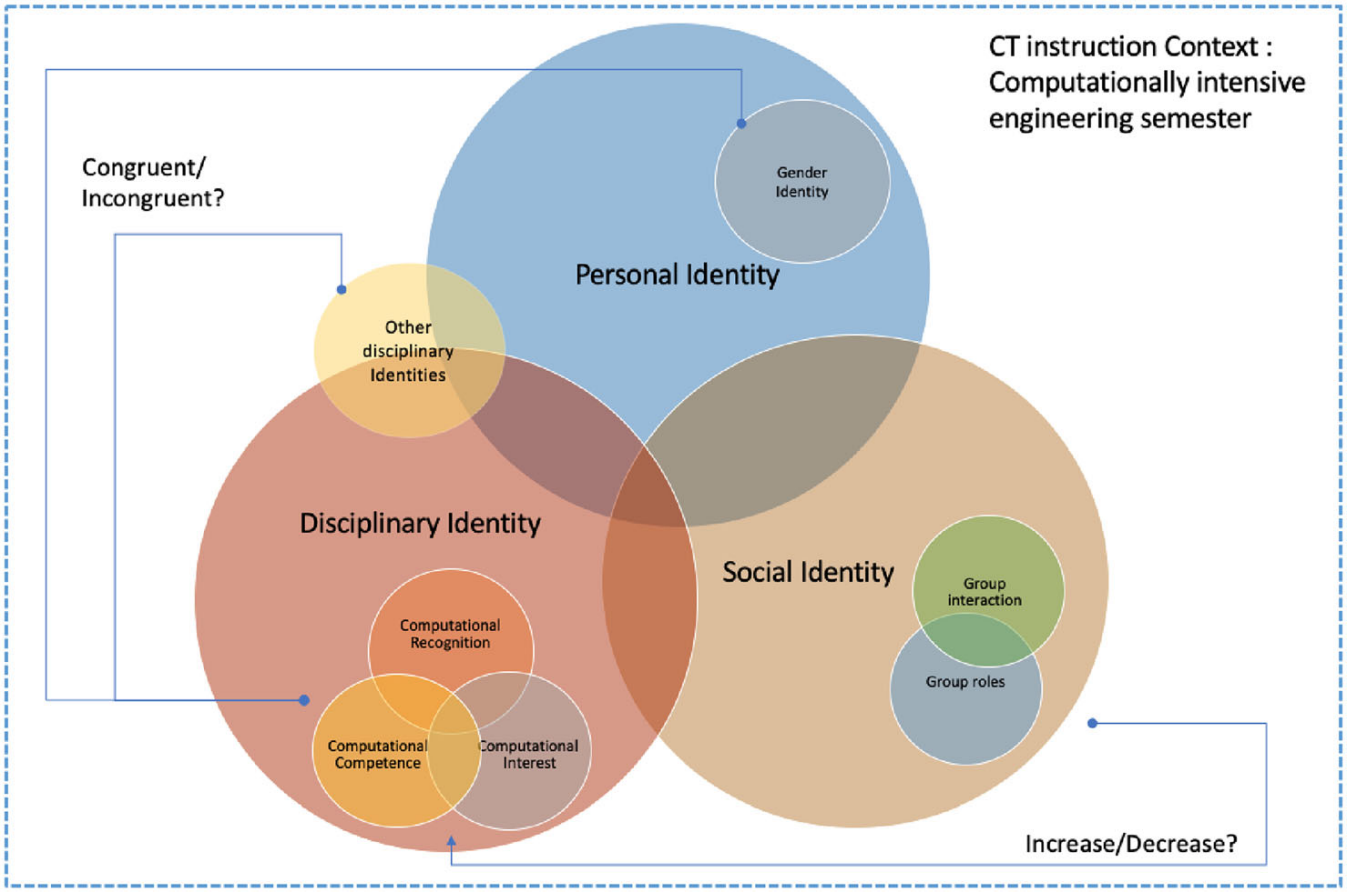
Framework for investigating computational identity.

For the current study, the *disciplinary identity* space is focused primarily on the student’s computational identity, but we also recognize that the students are likely developing engineering identities. The *social identity* space incorporates the social interactions like group work interactions in the classroom. Finally, we include *personal identity* to consider the student’s self-identified gender. The research questions are constructed based on this framework which aims to understand students’ computational identity development, the congruence of computational identity with other disciplinary identities, and congruence of computational identity with gender identity. The present study focuses on the relationship between disciplinary identity space and the personal space as shown in Fig. 2. Our investigation of social identity is presented in the first author’s dissertation^43^ and a second paper currently in revision.

## The Present Study and Methodology

Qualitative approaches are typically used to study sociocultural interactions because these approaches help the researcher(s) understand the interactions and experiences from the participants’ point of view. Qualitative methodology is often used in discipline-based identity research,^3,10^ particularly when new frameworks are being developed or the research examines identity development in a new area. To investigate the computational identity of undergraduate engineering students, this study is designed from a sociocultural theory of identity perspective. This study was guided by three related research questions:

A. What are biomedical engineering and agricultural & biological engineering students’ conceptions of what it means to be “computational”?
B. How do biomedical engineering and agricultural & biological engineering students perceive their computational identity during an intensive computational course?
C. In what ways is computational identity congruent or incongruent with other identities students hold?

We use the theoretical framework presented in Fig. 2 to develop a semi-structured interview protocol for individual interviews with undergraduates. This approach helped with understanding the multiple, constructed realities of the participants in the context of a computationally intensive thermodynamics class for Biomedical Engineering and Agricultural & Biological Engineering students (while our focus is biomedical engineering and biological engineering, at our institution biological engineering students and agricultural engineering students are collectively considered “agricultural and biological engineering” students). The study included students who participated in the class in the Spring of 2020 (the 2020 cohort) and students who participated in the Spring of 2021 (the 2021 cohort).

### Semi Structured Interviews

A semi-structured interview protocol (see appendix) was constructed, with questions mapping to the main constructs of the theoretical framework: recognition, competence, and interest. Participants in the 2020 cohort were asked questions related to computational experiences, teamwork, and project roles. Each interview lasted between 40 to 65 min. Relationships between engineering and computational identity were investigated in the 23 interviews from the 2020 cohort. Five additional interviews were conducted with students from the 2021 cohort to further investigate the relationships between engineering and computational identity. While conducting the interview, the interviewer wrote memos which noted each participant’s pronouns; the participant’s reported age; the real and preferred fictional name provided by the participants; interesting points of observation during the interview with time stamps and a description of what the participant was wearing. The interviewer included notes about the participants’ attire because clothing can, at times, be an external expression of identity. Later in the paper we discuss gender stereotypes and share an example of how participants also saw clothing as relevant for external recognition as a computational person.

Participants' actual names, demographic information, and audio responses were kept confidential and transcribed immediately to protect participants' confidentiality. The interviewer personally transcribed the audio recordings of the interviews. When transcriptions were completed, the interviewer went through the recordings again to check that the transcripts were accurate. Seven interviews in the 2020 cohort were conducted online with Zoom when instruction shifted to remote learning because of the COVID-19 pandemic. These online interviews followed the same interview questions asked in physical setting-based interviews and were audio-recorded.

To de-identify the interviews, the real names of participants were replaced by the pseudonym provided by the interviewee. Students were told about the confidentiality of the audio recorded data before they began the interview. After the interview was completed, students signed a human subjects log, which had the student’s real name, duration of the audio recorded interview, and compensation amount approved by the university’s Institutional Review Board.

The data collection process described above was designed for collecting a detailed description to communicate the context within which the computational identity formation process took place and the goal of understanding behavior from the participants' frames of reference.

### Study Context

Participants for this study were recruited from a thermodynamics course offered in the agricultural & biological engineering and biomedical engineering departments. The thermodynamics course was chosen as the research site for two reasons: the specific focus on Biomedical engineering and Biological Engineering and the recent curricular revisions made by the instructors. These two rationales are described in more detail in the next paragraphs.

The instructors teaching the thermodynamics course revised the curriculum of the course by introducing computational modelling in the course. Specifically, they developed and implemented Jupyter Notebooks (an open-source web application that provides an interactive online environment for computing in the Google Collab interface). In each lecture, students were given a link to an online worksheet that contained the lecture notes as well as the computing exercises for that class period. The three class projects and exams also relied on the Jupyter notebook framework that has built-in python capabilities. Early in the course, students completed tasks like modifying an example script to change the inputs or to add new variables to a system of equations. Later in the semester, when the students had several examples to draw upon, they developed models from scratch. Modeling activities aided by computation provide a sandbox in which students iteratively developed predictions/hypotheses about how systems work and developed intuition about the function of thermodynamics systems. In the syllabus, ‘computational’ is used to describe the activities the students will be participating in and also outlines how computational modeling in Python will allow students to iteratively test thermodynamics models. More detail about the course, including the course syllabus, is included in Shoaib.^43^

The instructors were also interested in better understanding their students’ experiences with engineering and computation, partially to understand how the curricular changes may have impacted the students but also because they were generally interested in understanding their students’ experiences. This combination of recent curricular changes combined with instructor interest in better understanding students’ experiences motivated a larger study of computational identity in the context of the course, but also allows our team to better connect the findings from this study to instructional practice. However, while the instructors were interested in the research, the interviews were conducted by a graduate research assistant who did not have any instructional authority for the class. The course instructors did not have access to any identifiable information, including information about which students participated (or did not participate) in the interviews.

The research was conducted over two years with two different cohorts of students attending the thermodynamics course in the spring semesters in 2020 and 2021. The course had four major computational assessments during both offerings. Due to COVID-19, in 2020 the instructors had to revise the second half of the course. To limit students' in-person interactions, the course content was shifted to an online medium, and assessments three and four were assigned as an individual project. In 2021 all four computational assignments were assigned as individual projects.

### Participants

**Figure 3:**
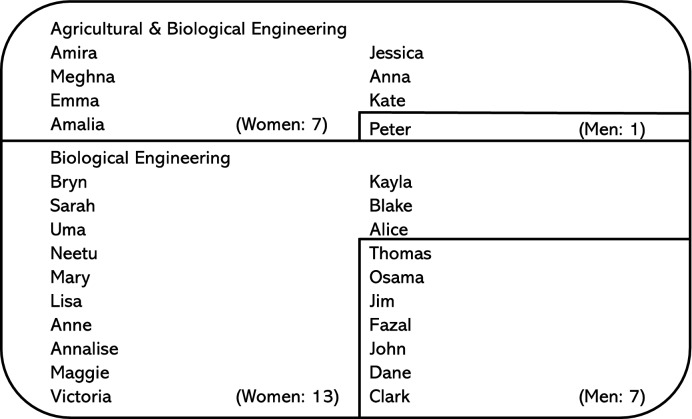
Interview participants.

Twenty-eight undergraduates (20 women, 8 men) majoring in either agricultural engineering, biological engineering, or biomedical engineering at a large midwestern university in the U.S. enrolled in a sophomore thermodynamic class participated in interviews. Participants (Fig. 3) volunteered for a 1-h semi-structured interview to share their engineering and computational experiences. Before starting the interview, the students were asked to share their preferred pronouns. This information was used to record student gender. Twenty-three participants were interviewed during the spring 2020 semester. An additional five participants, Kayla, Blake, Alice, Dane, and Clark were interviewed during the spring 2021 offering of the thermodynamics course.

### Data Analysis

Interpretation of the interview data began simultaneously with data collection (Hatch 2002, p. 179). The interview data analysis continued with the researcher's review of each transcript before member-check and redaction. The coding of transcripts was supported by MaxQDA 2020 (VERBI Software 2019) for data analysis. The analysis for the initial codebook construction began with selecting a sample of three interviews chosen to represent a rich and diverse set of participants' data.^41^ The criteria for the selection of these initial three participant interviews and subsequent interviews selected were:

- Researcher's judgment of the richness of the interview.
- Difference in computational experiences.
- Early (week 2), middle (week 8), and late participation (week 16) in the interview during the semester. (One interview was selected from each phase to construct the initial codebook)
- Gender variation.

During the first round of coding we developed the initial codebook by identifying connections between participants’ statements and aspects of the theoretical framework (e.g. expression of interest, instances of recognition, students’ perceptions of their performance, comments about students’ gender identity). We did this through a line-by-line analysis of participant transcripts^41,44^ where the code assignment was inductive as well as deductive (see Table 1 for an excerpt from the codebook). This phase consisted of at least two passes through each transcript, each time focusing on a different form of coding as deductive (based on the theoretical framework) and inductive (*in vivo*). The deductive coding meant coding for constructs and sub-constructs based on the theoretical framework in each of the participant's sentences.* In vivo* coding used participants' own words as codes. Then, axial coding was used for theming the data. Axial coding is used as a second-round coding technique. Axial coding helped to combine related codes found during first cycle coding* via* inductive and deductive thinking, which was necessary for drawing novel understanding.

**Table 1:** Excerpts from the codebook.

*In vivo* description	Initial codes	Secondary codes	Description	Construct
" I'm bored of programming; I feel like it’s the same thing over and over again there is other stuff that I want to do, and I want to try out " “So, you know normally I am a very lazy, procrastinator person. But it depends on my interest if I want to be efficient or not. So sometimes yeah, I am a computational person other times no I’m good, you can do it yourself.”	Boredom from programming Perception of self: lazy, procrastinator	Interest	How much attentiveness or not an individual show towards computational activities like programming or mathematical modeling.	Deductive code Interest^15,30^
“I would say computational person is somebody who is probably good at math and by being good at math they’re probably good at logic and they’re very focused on getting the calculations right.”	Perception of others: Good at mathematics Logical proficiency/thinking	Mathematics skills or mathematical modelling (Yes/No)	Ability to create a mathematical model of a given problem statement, or mentions proficiency in logic and reasoning	Deductive code Performance/ Competence^15,30^
“I think the technical aspect might come in girls leaving this discipline, because maybe a lot of girls have like grown up thinking like math is not something, they're supposed to be good at or can be good at.”	Perception of others: women have doubt on math ability Women not good at math	Women/Feminine stereotype	A thought about how specific types of individuals behave or can behave	Stereotypes (inductive)

## Findings

### Students’ Definition of Computational Identity

During the interview, the participants were asked to define a computational person, and then asked if they saw themselves as this computational person or not. In situations where the participants answered yes or no, they were asked to describe why they answered yes or no. In cases where they reported partial or moderate self-identification as a computational person, the participants were asked what would make them feel more like a computational person. It was important to understand how students define a computational person to see if there are any gender or media stereotypes associated with the emergent defining themes. Thematic analysis was performed on student responses to the question “who is a computational person?” to find the emergent themes based on student definitions. The students not only defined a computational person as having types of skills common to coding, but also discussed various skills as well as knowledge, abilities, and orientations to learning in order to be computational. In the following sections, the themes correspond to how students define a computational person.

#### Theme One: A Computational Person is Perceived by the Participants to be Proficient in Mathematics, Programming, and Problem-Solving Knowledge, Skills, Abilities

The first emergent theme refers to the knowledge, skills, abilities, and ways of thinking a computational person possesses. Twenty interview participants described a computational person as having mathematical, computer programming, and problem-solving skills, and abilities. Participants explained that a computational person has the ability to critically think through problems which are either mathematical or computer programming based. These responses indicate students’ beliefs that a person needs to have these skills and capabilities to have a computational identity.

For example, Jim (BME) started with defining a computational person as someone who has enough competence in mathematics, logic, and programming: “Well if I was to define a very computational person, that is like dealing with numbers, or dealing with logic.... If they are competent enough with obviously numbers, math, programming, that sort of thing.” In this example, Jim initially talked about a computational person in terms of the types of things a computational person does “dealing with numbers or dealing with logic” but Jim quickly shifted from the things a computational person does, to a computational person’s competence or proficiency.

In contrast to Jim’s definition, Kate (ABE) did not include the type of things a computational person does; instead, she focused on the set of skills and proficiencies she associated with a computational person. Kate started explaining about a computational person based on mathematical skill sets followed by programming proficiency she said “I would say computational person is somebody who is probably good at math and by being good at math they are probably good at logic and they are very focused on getting the calculations right. Are probably very detailed oriented. There is mathematics and programming. I would say a computational person is like a logical, they know how to work with like different technologies, and they are kind of like the phrase ‘techie’.” Kate identified a set of skills related to math and working with technology, as well as modes of thinking such as ‘logic’ and ‘attention to detail’. Interestingly Kate, herself viewed these different skills as inherently connected, as she notes ‘being good at math [suggests that a person] is probably good at logic’.

Lisa (ABE) also focused on skills and abilities without being prompted to express her views on skills and abilities. Lisa, like Kate, identified mathematical skills as a core part of a computational person and expressed interest in mathematics, “I have always enjoyed math and I think I am a strong student in mathematics, but I think I am only like the tip of the iceberg with computational problems”—notably going forward she focused on the practical application of these skills in a problem solving capacity,“ I would think a computational person is someone who, not only succeeds in mathematical problem-solving type problems, but also someone who applies that to like their everyday life or other problems that they are solving. Someone who thinks more with numbers and going through that process, that is how I would describe a computational person.” For Lisa, a computational person is not only proficient in certain skills like mathematics, and problem solving but also possesses the ability to apply these skills in practical life or real world.

For most of the interview participants, a computational person is someone who has mathematical, computer programming, and problem-solving skills. Some of the participants recognized this as a combination of the things a computational person does as well as the competencies they have; others recognized relationships between these skill sets and recognized associated skills and attributes, like being detail-oriented and being good at logical thinking. Several students also described having the ability to apply these skills to practical life and other fields of study.

Based on the literature, three primary sub-constructs that contribute to the development of a computing identity are belief in one's performance/competence, interest, and recognition in computing. Students were not prompted to define a computational person through skills or abilities, yet they defined a computational person through sub-constructs by explaining which skills the computational person performs and excels at and the interests of a computational person. Participants discussed not only having skills and abilities but also ways of thinking. This finding aligns with the trait-based stereotypes in STEM e.g., STEM is for geniuses (Starr 2018). However, participants did not express how the computational person was recognized by others as part of their definition of a computational person, which was not consistent with Mahadeo * et al*.'s^30^ research.

#### Theme Two: Participants’ Shared Belief that a Computational Person Possesses an Ability to Make Rational Decisions When Working on a Computational Task

The second emergent theme found from participant explanations was that they saw a computational person as someone who considers possible outcomes and consequences in the process of making decisions. Participants directly addressed that a computational person can make rational decisions. Uma and Jim reported detail on why a computational person is competent in making decisions.

Uma (BME) connected the ‘logical’ decision making of computational people, with practice of programming, “I would describe a computational person as someone who thinks very technically, someone who looks at the consequences of like what they do, like thinking ahead. Because when you are coding you have to always think about like if I do this, what will happen then? That's what I think of when I think of a computational person.” For Uma, the ability to think ahead gives a computational person the ability to take an appropriate decision by prediction

Jim (BME) gave explanation on similar notes as Uma about a computational person’s decision-making skills. According to him, a computational person makes rational decisions. He says, “a computational person has ability of decision making based on weighing pros and cons of different decisions and then going with the least damaging decision I guess when I think of a computational person, I think of someone who I wouldn't say just think things through, but like think things logically and when it comes to making decisions, they weigh their options in a logical way as sort of like statistical way. Like for me it's like if I was to make a decision, I would think, Like, Oh, there is 75% chance this decision will go wrong and we don't want that happening, so I am going to take the other option, which might be 30% failure sort of thing as compared to 75%. That's specifically decision making.” For Jim, a computational person will take decisions which guarantee highest probability of success.

For these interview participants, a computational person is someone who has skills of rational decision making. The participants shared a belief that the ability to make decisions informed by computational work is essential to have a computational identity. This is connected to the theoretical framework through the competence component where competence in this case is the ability to succeed in a computational task by using the skill of decision making. All the students who described this “logical” decision-making process in computational people, made positive value judgements. They considered this to be essential to “good decision-making” indicating that all of these students had been socialized into computational ways of thinking even if they had differing levels of self-identification as a computational person.

#### Theme Three: Translating Computational Problems to Code Using Computational Thinking Processes like Pattern Recognition and Decomposition

Five participants set forth in detail that a computational person can translate a given computational problem to a computer program. Neetu and Allison emphasized having the capacity to produce a computer program from a given computational problem is essential to being a computational person.

Neetu (BME) talked about having the ability to translate a given computational problem to programming. She explained that the computational person performs this translation by keeping the perspective of the machine in sight by understanding how the natural language can be decomposed for the machine. She says “Generally, a computational person to me is someone who can look at a problem and understand how to phrase it so that a computer could understand it. Can you look at a problem? And see, okay, here is how I need break it down. Here's all the pieces I need to take apart. Here are the parts that need to become loops. Here are the parts I need to define. And this is how I am going to lay it out. Someone who knows how do you do that? Versus like someone who cannot do that, is the basis of a computational person to me”. For Neetu, having the competence to decompose and then lay the decomposed pieces into a translatable pattern for computer programming is essential to being a computational person. Allison talked about similar skills and explained, “someone who can like take a big problem and break it down into small steps and understand how that works.”

Some of these participants mentioned having a particular skill of translating computational problems to code. Otfers described additional abilities associated with translation between these languages as being comfortable and knowing how to decompose as well as recognize a pattern, which is connected to the competence construct of computational identity development. Because competence is the self-belief to succeed at performing a specific task or skill, when a student believes that they have the skill to recognize patterns in computer programs or break down a problem into multiple parts they feel they are competent at pattern recognition or decomposition.

### Students’ Description of Their Own Computational Identity

This section pertains to how students described their own computational identity or related themselves to their definition of a computational person. The participants were asked an open-ended question “do they see themselves as the computational person they just described?”. Participants gave a response of “yes,” “no,” or “partially”/“moderately,” and were prompted to explain their response. The participants were sorted based on their response to understand how students describe their own computational identity.

### My Computational Identity is in the Making

It was no surprise that 20 of the 28 interview participants responded that they partially or moderately adhered to the computational identity because the definition of a computational person is very complex and involved having many skills, traits, abilities, and ways of thinking. They have skills (logic, programming, computational modeling) that are essential to this identity; however, they are working on the missing pieces of becoming a computational person.

#### Men’s Self-Perception of Computational Identity

Men in this study, on average, reported that they are proficient computer programmers and very comfortable in performing computational activities. Here are excerpts from Thomas, John, and Peter.

Thomas (BME) started coding early in life and joined the Midwestern university with an interest in computer science and engineering. He had a decade long programming experience and showed passion for programming because he taught a programming course earlier. When I asked him if he feels like he is a computational person or not he replied by saying “yes and no. I am good at coding and I like it…. But the thing is I am not very detail oriented and my issue is that I cannot be because I am so ADHD, skip around, I tend to miss the small details.” Even though he had skills associated with the computational person he defined, he was only partially adhering to the computational identity.

Another student, John (BME), had previous extensive experience of programming and came into the Midwestern university with an interest in computer science and biomedical engineering, just like Thomas. John partially adheres to the computational identity. He believed that a computational person is very planned and logical. He explained why he feels that he has this partial identity because “I don’t really walk into something with a definite plan. I am kind of a person who have plans but also half kind of wings my way through life. So, I think that’s like a different approach because mine is more half and half and [a computational person’s] is more like wholly based on just step by step and logical.”

Peter (ABE) was confident about his computational skills and competence. When asked about if he adheres to a computational identity, he answered “if there was a spectrum, I am kind of in the middle. I think looking at it in terms of spectrum where you can be more towards a computational person rather than just being specifically computational is a beneficial way of thinking about the question. Because if you sit down and all you do is you look at numbers, you do not necessarily think it through, you are instead relying on the code. But what if the numbers you are receiving don't make any sense. Now you have to think outside of the box, and you can no longer rely on these computational models that I have been working on.” He responded positively to how he thinks his computational identity is in the making and he is working towards improving it to reach the higher end of the computational identity spectrum he mentioned.

#### Women’s Self-Perception of Computational Identity

Women participants in this study reported experiences and instances that inform their self-perception of their computational identity. Amalia (BME) was not confident about her programming abilities and says she is not certain if she is a computational person or not. She was undecided because of some negative academic experiences with the instructors and male peers “I was the only female on my table, and I felt very out of my comfort because all were male, and the majority had computer science experience. The great majority. I had absolutely zero and I had no clue what we were doing. I felt the professor did not facilitate for people who had never even touched any computer language. I hadn't even written a program like print hello. You know? that was kind of a bad experience for me. And at that point I was like, Oh, I am not going to be a good computer person. I am not good to go as a good programming person and this is not my thing. I am very hardworking and very open minded. So, I was like, you know, I am making my thing. I would just learn. About being a computational person, I don't know. I think I have some valuable, computer science skills that I don’t think I am the best nor the one worst.” Amelia’s negative experiences with instructors, peers, and not being able to be introduced to computational resources early in life lowered her programming ability self-efficacy and made her feel like she does not belong,^39,45^ however she had a positive attitude toward working persistently to become proficient at computational skills.

Kayla had her first experience with computer programming at college. She expressed her interest in computational tasks; however, she had difficulty with using the programming language because of being a recent starter, “I don't like doing computations in programming yet. I'm getting there, but I do like doing computation”.

Sarah (BME) similarly mentioned not being introduced to programming earlier in life as “I think that it just depends on like how much technology people are exposed to previously in life. Like I had no experience, I feel like if I was used to it, I would be interested in it faster genuinely”. Sarah connected high interest in computational activities with having early access to computational resources.

When comparing women and men students who report having a partial computational identity based on interviews and surveys, women were consistently lower in their sense of computational skills because of lack of experience. The contribution of a lack of prior experience to a lower sense of computational skills in women is consistent with explanations proposed by Goode* et al*.,^18^ for why and how female high school students are attracted into the field of Computer Science or not. They found that there were few learning opportunities at the high school level, and that pre-set definitions of interest play a key role in shaping choice.

Students reported that they have a partial computational identity because they possess some of the traits associated with their own definition of a computational person, but not all the traits. However, responses suggest that students’ partial computational identities are still developing, and women were more determined to work on building their computational identity.

#### I Have/Don’t Have a Computational Identity

Meghna (ABE) worked in a male majority project group as a technical leader of the team. She depicted a strong sense of computational affiliation with computational activities. She shared that “I identify as a computational person. I always have that starting trouble because when you haven't touched a language in a while, it's just you have to remember all the syntax, you remember and once you get it down then things just start flowing in and you are starting to build and build your phone. So, I now enjoy it more than I did when I was younger.” When asked about her ability to do computational problem solving, she went further saying, “I definitely feel like can do well. There's a lot of resources out there. if you don't understand something do, you can always look online. You can figure out how this works. There's a lot of information surrounding it. I definitely do not see it, why I would not be able to do well in it.” Meghna had a positive outlook towards her abilities to perform well on computational activities to develop a computational identity.

While Meghna reported working on developing her computational skills, Bryn (BME), on the other hand, was very clear on why she does not view herself as a computational person. The reason she gave for not feeling like a computational person was through comparing herself to others based on prior programming exposure. She expressed “I do not really see myself as a computational person. Last time in the project I did do like a good portion of the coding. I feel like coming to college has definitely increase my coding skills. I came in with no coding classes, no coding experience because my high school did not offer anything like that. So even though I've definitely gotten a lot better and I have definitely got better at solving computational problems, I don't really see myself as that person just because I think there are so many people who have more experience or have more confidence or more abilities in general. So, I guess from comparing myself to other people, I don't really see myself as that computational person.” Despite improving her computational abilities in college, when she compared herself to other peers who were more proficient at programming, she could not see herself as being a computational person. Bryn attributed her struggle to internal reasons where she felt that she was not at the level of her peers. This finding is consistent with LaCosse* et al*.^26^ who report that the attribution of failures for women in STEM are associated with personal views of capabilities rather than external factors. The attribution of their failures or struggles to capabilities rather than external factors creates a disassociation in computational identity.

### Congruence Between Computational Identity and Other Identities

During the analysis of interview transcripts to answer the second research question, the various identities described by students were coded and used to determine the level of congruence or incongruence between these identities and computational identity. The findings emphasized which other identities (i.e., gender, engineering, etc.) the participants perceived as compatible or incompatible with the computational identity and why. The focus of the analysis looked at the balance between these identities rather than the switch between roles. The three major identities the participants linked to computational identity were gender, engineering, and creative identity. The findings from interview data are complemented with classroom observations and survey data. The findings pertaining to the second research question are divided under the following sections associated with gender, engineering, and creative identities.

#### Gender Identity and Computational Identity

"Gender identity" has a strong presence in our lives because, generally, it is biologically and socially imposed.^14^ Stereotypes and dominant images of gender and the tasks associated with gender surround us all the time in all societies and cultures because it is typically a social construct. The participants’ personal construction of their gender (determined through self-identification of their pronouns) is used for this study. This section provides an understanding of how students describe the relationship between gender identity and computational identity to understand the congruence between these identities and how they perceive their peers’ and their own abilities to adopt these identities. Participants were not asked a direct question about their gender identity in relation to computational activities or a sense of belonging. Rather, participants were asked an open-ended question, "Would you like to add something on being a (gender of participant) in biomedical or biological engineering, which is becoming more computational day by day?" at the closing of the interview.

#### Gender Stereotypes and Experiences of Uneven Experiences with Computational Tasks

Most of the women participants talked about their experiences of gender stereotyping where they felt that they were viewed as less capable than men at performing computational tasks. This strong feeling that their contributions are not appreciated makes them feel like they are at a disadvantage when it comes to computation and developing a computational identity. Amelia expressed how she feels that her feminine practices come in conflict with being perceived as a computationally proficient woman and expressed views of what a computational women stereotype is (described below). Anna conveyed how women are less confident about their computational abilities, and Sarah shared how women take up more of the non-technical work in the classroom on computational projects.

Amelia (ABE) explained that if she was too overtly feminine, she would not be recognized as a computational person. She mentioned recognition, and she explained that if she conformed to more masculine norms she might be recognized as a computational person. She says, “I used to see if women looked [like] women, they do not have time to do anything else. They do not know how to code [because they spend time to look feminine]. Like people are very split. When they look, if they look at a girl who has a tee shirt with an anime character and like and a NASA key chain, they would probably think she can code. Whereas when they look at a woman who wear heels, her purse and makeup on. They think she can't code. I don't know why, but it happens.” For Amelia, appearing to be more tech savvy (wearing an anime tee shirt, having a NASA keychain) is more aligned with the socially recognized image of a computationally proficient person. Like Amelia’s explanation, Berg* et al*.^4^ reported that the stereotypical image of the computer scientist that children had, was incompatible with that of their stereotypical image of a female due to a gendered view of computing as being a male discipline with a lack of female role models. These findings are also complementary to the research of Young* et al*.^52^ on how masculine nature nerd-genius stereotypes affect women’s motivation and STEM identity.

Some women participants also talked about experiences of gender differences in experiences with computational tasks during engineering team experiences. Sarah (ABE) mentioned instances where she felt like women engage in tasks like report writing which is not as computationally intensive as programming. She says, “I remember making this note to my engineering team in second semester. I said “do you notice that all of the people that are doing most of the coding are the guys in the group and then all of the people that are mostly writing the reports are the girls. I just want to know why this happens?” she took a pause and looked down and after a heavy breath she said “I really do not understand because we all here want the same thing. But that's just one like it falls back to, and it was so crazy for me to look around the room and I am seeing this myself and even myself, I was falling into that role. I was writing the reports and I think it is kind of just being an engineering, you (women) are already kind of like breaking a standard that's set.” Sarah may have been referring to the fact that by being in engineering, she is already breaking a standard. To actually work on technical tasks like programming, she would have to break an additional standard. She went on saying “I do tend to be a person where I am like, if they (men) want to code and they (men) are good at it, how could I deny them to do that? And then, you know, I just be more complacent when I can tell they (men) do not want to explain it to me, to that I'll just be like, okay.” Goode* et al*. also show that in classroom environments, women students who take Computer Science have adverse experiences, where greater technology experience in men and alienation of women are part of the cultural environment. When it comes to role allocation our findings were consistent with Meadows* et al*.^32^ that women typically not only take the least technical roles, but they are also less likely to acknowledge this gender bias. When seemingly unimportant or supporting roles are assigned to reflect the social stereotype of men in engineering as experts and women in supporting roles, women like Sarah may feel unvalued by the majority.^32^

#### Interests, Capabilities, and Gender

Interests and capabilities are independent of gender. Interests and capabilities are frequently associated with gender. In the interview with Meghna (ABE), when asked about her interest and proficiency in computational tasks being a woman, she described that her computational interests are something that can’t be cultivated alongside women’s friendships “I noticed very often that the skills I like, they do not help me fit in with other people in my gender. I picked things like learning video games by just hanging out with my guy friends. It's just I wish I had met more like-minded females at a young age. I wish I did not want girlfriends as again I wanted in childhood, because as a kid, if you want friends. I am a girl. So, my friends should be girl. Right? So, it is just that if I had thought I am a person, I like these things. I want friends who like these things. Game changer! I would not have cared as much that I did not have a single girlfriend. At least I had people who like similar things and interests.” She described how she has interests that align with the interests of a computational person, but when she was younger did not find other girls that shared these interests. In order to become friends with the girls, Meghna would have to minimize her interest in chess or programming. She realized that to fit the feminine norms of the girls’ group she would have to let go of her computational interests which depicts an incongruence between feminine and computational identity in Meghna’s case.

Mary (ABE) expressed during a follow up response to a prompt question that people tell girls to pursue coding when people think the girls will need the skills, but not because they think girls would be interested in it. She says “I don't know if it's necessarily that I am a woman, but I feel we don't do a lot of computation just by nature. I have all my friends who are really into coding outside of class all happened to be guys. And I don't know if that's a coincidence or not it could be, I don't know. it is hard because sometimes it feels like there are not opportunities, but like girls aren't really like encouraged to take on these extra like challenges for coding until you decide that it's a part of what you want to do. Like for example, in high school, I never really felt like anyone encouraged me to look more seriously into coding until I told people I was going into engineering. That's when they (instructors) started recommending coding related stuff to me. I don't know it's not that there are no opportunities, but it feels like girls are not pushed to like to pursue them until it becomes relevant to them. Like no one tells a girl to go, oh you should look into coding. Because I think you would actually enjoy it, but you should look into coding because you're going to need it later.” Based on what Mary reported when her instructors recommended to practice programming because she was going into engineering, she felt that programming is introduced as a skill rather than an interest to women. Interest is a subcomponent of computational identity based on the computing identity framework. If women engage in thinking that programming is a skill rather than interest, they would not be able to grow interest in computational activities.

#### Engineering Identity and Computational Identity

Some participants talked about engineering identity and their computational identity in follow up questions. When Emma (ABE) was asked a follow up question on why she thinks an engineer and a computational person are different? she explained at length, “Being an engineer is about what I was saying earlier about like applying your knowledge to make something new or make something better. But being a computational person, I would say it is applying like more of a mathematical knowledge. Make something better. I guess I see engineer as designing what needs to be done and then computational person is like implementing that plan. An engineer's going to say, okay, we need to figure out a way to make this run this much faster. And computational person will say, okay, these are all my variables and I need to manipulate them all so that I get the right output.” For her an engineer is the visionary and the computational person is the practitioner, they work with each other to be successful which brings out the congruence.

Similarly, Maggie (ABE) expressed how engineering and computation are intertwined and to be a successful engineer one must be proficient in computation. In a follow up question when asked if she thinks that being an engineering person and a computational person is similar or different? She replied, “I am having a hard time to differentiate the two because I feel like you need to be a computational person to be a successful engineering person.” For her and Emma, being computational was inherent to be a successful engineer.

Kayla expressed that most of her experiences in engineering have involved computation. She said*,* “I would say for the most part engineering and computation are intertwined. maybe it's not thermodynamics specifically, but, I guess it's just the experiences that I have had so far. I'm in engineering classes, so my projects are engineering projects and they all involve computation. I feel like all engineers must do computations. I feel like not all engineers enjoy doing computations”. For Kayla and Maggie, engineering and computation were intertwined.

While Kayla talked about the co-existence of engineering and computation, Allison differentiated between the two. She expressed how engineering was more innovative and creative than computation. She said, “I think with engineering, it might be looking at a problem and being able to bring a creative aspect to it. You can be creative in coding, but you know, engineers must improve upon things or check things to make sure they are okay. quality engineers, for example, I feel like that's more computational just because they already have like a list of things that need to be a certain way. And if they're not, then they have to produce a new solution and that's engineering based, but computational is taking something that exists, breaking it down, making it like understandable in a different way vs. engineering is like creation.”

While computational identity is congruent with engineering identity for many respondents, as these examples point out, students often have a more nuanced view of engineering identity that really relies on creativity and creation. This perspective, while not dismissing the role of computation in engineering, provides a more holistic view of engineering.

## Discussion and Conclusion

Prior research provides limited findings related to how students define a computational person. In the previous literature used for the theoretical framework of this study, researchers looked at computing identity by identifying three sub-constructs: interest in computation activities, competence in performing computation activities, and perceived recognition as a computing person able to perform computing-related activities.^15,30^ Our aim was to understand if students describe having computational competence, recognition, and interest as essential to having a computational identity in their definitions of a computational person. The following three themes emerged based on student definitions:

1. A computational person is proficient in mathematics, programming, and problem-solving knowledge, skills, abilities, and ways of thinking.
2. Participants shared a belief that a rationalistic, analytical, and linear decision-making approach is essential to having computational identity.
3. A computational person is able to translate a given computational problem to a computer program through pattern recognition and/or decomposition.

The students defined a computational person through skills and abilities which correspond to the *competence* construct and perceived *recognition* to perform computational thinking related activities of the theoretical framework. A computational person is recognizable by not only the skills and abilities to perform computational tasks, but also by how they perform computational tasks. None of the students described a computational person as having *interest* in computational activities in their responses. However, the students did express their own interest in skills they associated with being a computational person. Based on the emergent themes found from interviews corresponding to the definition of a computational person, a computational person is complex and is perceived to possess many abilities above and beyond just the technical knowledge of computation. It was noted that most students said their computational identity was in the making. When asked what would make them feel like they were a computational person, they responded that a certain skill or ability was missing, or that they were working on improving a certain skill or ability. Given the complexity of how students are defining what it means to be a computational person—with many different facets that overlap engineering, computer science, and math, it is not surprising that the students in our study did not feel competent and confident in every skill and ability associated with being a computational person.

The students who either completely adhered or did not adhere to the definition of computational person had very narrow views of the skills, and abilities a computational person will possess. Some students held negative perceptions of “a computational person” such as associations with the nerd stereotype where a computational person engages in excessive computer programming or making decisions just based on statistics and not involving human intuition in the decision-making process, or that the computational person is emotionless.

### Congruence Between Computational Identity and Other Identities

Previous research reports gender stereotypes in computing disciplines (e.g.^33,38^) as one possible cause of the underrepresentation of women in computing disciplines. Participants were asked an open-ended question of their views on gender and Biomedical Engineering and Agricultural & Biological Engineering becoming increasingly computational, as well as questions related to group interaction to understand the congruence between gender and engineering identity. Participants' experiences of group interaction and team roles based on students' gendered experiences were analyzed during sub-theme generation. The codes related to these experiences were connected to generate relevant themes. Women participants reported incidents of gender stereotypes and unequal division of computational tasks.^43^

If someone sees a domain or role as congruent with self, they can easily develop an identity in that domain. When incongruence happens, it causes identity conflict that can lead to coping strategies to minimize one's identity in a particular context to create fit or to leave. Many individuals are likely to be incongruent with the characteristics of the nature of tasks they perform or the people they engage with. Computing being a masculine discipline presents these stereotypical characteristics, and both men and women who want to depict acceptance in this masculine field adapt to these characteristics.^1,12^

Stereotypes are a major problem that affects many women who want to pursue computing as a discipline. As in many other sectors, technology stereotypes are integrally related to much broader cultural gender stereotypes.^37^ Best* et al*.^5^ suggests that the stereotypes of men are associated with categories like “rational, independent, egotistical, and unemotional.” A woman is described as be “affectionate, sociable, sensitive,”. Parallels can be found when these male stereotypes are compared with the characteristics of scientists. This contrasts with the stereotypical image of women, where women have tended to be seen as sociable and concerned.^5^ A mix of men and women participants like Fazal, Osama, and Ekta defined a computational person as someone who spends excessive time on computer programming, so they do not have time to socialize. They responded that they partially adhered to a computational identity and not fully because they engage in social activities instead of performing computer programming all the time. They had found their ways to be in congruence with the discipline without adhering to antisocial stereotypes.

Women participants who reported that their computational identity was in the making or reported having a strong computational identity found ways to maintain both their feminine identity and their computational identity. Women who reported having strong computational identity developed the computational identity based on coping and hiding strategies like adapting to tech norms for being recognized as a computationally competent person. Many of the women participants did not note any incongruences, and the men students did not talk about gender/gender congruence.

When participants were asked what it meant for them to be an engineer, some of the participants described having proficient skills and recognition in mathematics and engineering in congruence with computational identity. Seven participants described that an engineering and computational person has intertwined skills. Engineering identity and computational identity were found to be in congruence. This depicts having an engineering identity as in alignment with having a computational identity or vice versa in engineering students.

### Implications for Future Research

Further research is needed to investigate the incongruence between creative and engineering identities. The social disconnect may erode students’ sense of belonging, an important motivator. When students have to choose either between “the creative” or “the technical” side of engineering, it creates an incongruence between their identities. More research on merging arts with engineering can shift the incongruence between these identities into congruence.

The current study investigated student’s computational identity throughout one semester. A longitudinal study that tracks students’ computational identity development would provide useful insights for engineering education. Also, future studies might further explore biomedical and biological engineering identities more specifically (rather than broader “engineering” identities) to further investigate congruence and incongruence between computational identity and biomedical/biological engineering identity.

Student experiences of sense of belonging operate beyond the lens of gender alone. Intersectionality (i.e., race, ethnicity, class, sexuality, age, nationality, etc.^21^) among students’ different identities is an additional direction to consider. This would help us understand additional ways that students understand what it means to be “computational” and other ways students may or may not resonate with having a computational identity. For example, recent work suggests that Black women and girls may hold interest in science without developing a science identity,^49^ or see computer science as a useful tool without needing to develop a computer science identity to persist in undergraduate computer science programs.^28^ Additionally, our interview participants only identified as belonging to a binary gender. The experiences of participants identifying as non-binary or another gender identity can be very different, and additional research is needed to understand the computational identity of these other groups of students. Further, we note that our study included many more women than men. While this is consistent with our focus on understanding factors related to women’s participation in engineering, we recognize that additional research that included more men may have revealed additionally findings.

Finally, in our interviews we chose to leave the definition of “computational” open to students’ interpretations. We chose this approach knowing that students encounter the term “computational” in course syllabi, descriptions of graduate programs, and job descriptions—and that the way this term is defined can very. Leaving the term undefined allowed us to learn how students understand the term. Future research might investigate how students see themselves as a computational person based on a common definition provided to all participants.

### Recommendations and Summary

For most students, computational identity is congruent with disciplinary identity and it is “in the making.” Stereotypes and perceptions of what makes a person a “computational person” are common and enter into the team dynamics and role assignments in the team. There needs to be instructor intentionality to mitigate the stereotypes and biases from being detrimental to a student “in the making,” and in the formation of teams. We also found that students associate specific skills with a computational identity. This informs how an instructor can craft assignments to shift the focus to developing specific skills or tools related to computational modeling processes.

## Appendix

Interview Protocol for Identity, Computation, and Gender Experiences—Cohort 2020

### Opening Statements

Thank you for agreeing to participate in the study. I am a graduate student in Engineering Education, and I am studying the student experiences about computation, identity and gender in the thermodynamics classroom.

### Identity

#### Background

- What experiences brought you to engineering? (interest)
- What has been your experience as an engineering student? (ask them about good and bad experiences)

#### Present

- Do you engage with your peers in and out of class?—What is most challenging?
- Who is a computational person?
- Do you see/describe yourself as a computational person?
- How do you see this course contribution to this computational identity?
- Do you believe you can do well in computational activities?
- Which skills and competencies do you believe are essential to have as an engineering student?

#### Future self

- Can you talk about your future aspirations?
- Which skills and competencies do you perceive are essential as a professional engineering? (competence)

### Recognition

- Can you talk about how your peers and instructors view you as an engineering student?

### Computation

- What skills do you think are essential in your field of study?
  - Follow up—why do you think this “xyz” skill is important?
- Do you use programming concepts in your BME classes?
- How comfortable are you with programming or computational activities? —yes, why
- What previous experience did you had with programming before taking this class?
- What kind of new challenges are you facing with programming this semester?
- Does your team or peers help you when you are performing a computational task?
- How do view the role of computation in professional engineering?
- Can you tell me about your experience of working on computational activities? (these activities can be from formal or informal settings)

### Teamwork

- Tell me about your class team peers? Are they the same peers you work with in every class?
- Tell me about your role in the team?
- Tell me more how you work with your team?
- Tell me about how your peers view you in the team?
- Which member do you believe is more helpful and why?

### Persistence

- You talked about when you faced challenge while you were engaged in the computational experience…. Why did you keep going when it got really tough? (What motivated you? What tools or coping methods did you use to keep you from quitting?)
- Who were your peers as your worked through this process?
- What has been the biggest obstacle you’ve overcome as an engineering student?
- Give an example of a time when you had difficulty balancing your personal and work objectives. What did you do?
- What motivates you to keep working through the computational task?

### Conclusion

- Do you have any other thoughts on you being a “xyz-gender” in a discipline which is becoming more and more computational with time?

Interview Protocol for Engineering Identity, and Computational Identity—Cohort 2021

### Background

- What experiences brought you to engineering? (interest)
- *How do you feel about being an engineering student/your experience about being an engineering student? (new addition as a follow up question)

### Present (Identity in Engineering and Computation)

- Do you engage with your peers from thermo class?—What is most challenging?
- How do you describe yourself as an engineering student?
- Do you see/describe yourself as an engineer?"
  - Follow-up if they answer “degree”: How would you describe someone working for engineering company but has a degree in CS e.g.
- How do you see this course to be contributing to your development as an engineering/ what part of the course is helping you to see yourself as an engineer?
- Do you believe you can do well in engineering activities? (performance)
- Do you see/describe yourself as a computational person?
  Follow up questions
  - how those two things differ engineering vs computation?
  - Is a computational person a subset of an engineer?
  - Are all computational people engineers?
  - Are engineers more computational than the average person?

- How do you see this course to be contributing to your development as a computational person/ what part of the course is helping you to see yourself as a computational person?
- Do you believe you can do well in computational activities? (performance)
- Follow up questions (How comfortable are you with programming or computational activities? Or How do you view your programming/computational modelling abilities?)
- Do you believe that your “computational modelling intelligence” / ability to do well in computational modelling can change? (growth mindset)
  - Follow up: What would help you with change?
- (Follow up question about why you don’t think it can change or tell me more about the ways in which it can improve)

### Future Self/Content/Skills

- Can you talk about your future aspirations? / what do you want to do in your future work?
  Follow up
  - How do you see the content or skills learned in this class relevant to your future work)
  - Is there anything else from this class that you think is relevant to your future work)

### Recognition

- Can you think of any time when your peers or someone else asked you for help in a computational modelling activity/task?
- Can you think of any time when your peers or someone else asked you for help in an engineering related activity/task?

### Computation

- What previous experience did you have with computational problem solving/programming before taking this class?
- What kind of new challenges are you facing with computational problem solving/programming this semester?
- Are there any people you go to for help when you are working on a computational task?

### Persistence

- You talked about when you faced a challenge while you were engaged in the computational experience xyz why did you keep going when it got tough? What motivated you?
- What tools or coping methods did you use to keep you from quitting?
